# Cleaner, Happier, Healthier: Sesame Workshop’s Water, Sanitation, and Hygiene Intervention among Low-Income Groups in Bangladesh and India

**DOI:** 10.3389/fcomm.2017.00020

**Published:** 2017-12-11

**Authors:** Abigail R. Bickford, June H. Lee, Dina L. G. Borzekowski

**Affiliations:** 1 Department of Behavioral and Community Health, University of Maryland, College Park, College Park, MD, United States; 2 ELMA Philanthropies, New York City, NY, United States

**Keywords:** hygiene, handwashing, sandal wearing, children, media, latrine

## Abstract

This article evaluates a pilot intervention of Sesame Workshop’s “Cleaner, Happier, Healthier” media program promoting water, sanitation, and hygiene (WASH) knowledge, attitudes, and behaviors among vulnerable children and their families in impoverished areas of Bangladesh (*n* = 240) and India (*n* = 258). Raya, a new Muppet^®^ was developed and introduced, advocating for healthier WASH behaviors. As part of the intervention, two approaches to health messaging were developed framing WASH as a personal behavior (the “me” intervention) or a social endeavor (the “we” intervention). In each country a three-armed approach employed groups focused on the “me” and “we” interventions and a comparison group. Both the “me” and “we” groups improved in WASH measures over the comparison group; however, there were limited differences between the “me” and “we” groups. Target behaviors, such as using the latrine, wearing shoes, and handwashing, improved when examining change before and after the intervention, and the intervention was predictive of positive knowledge, attitude, and behavior change. Results of this work are limited due to lack of a randomized control trial but suggest that participants who received the “Cleaner, Healthier, Happier” interventions will be more prone to engage in healthier hygiene behaviors.

## INTRODUCTION

Globally, one in 10 deaths among children under 5 years is caused by diarrheal disease, resulting in around 800,000 fatalities annually (Baker et al., 2014). Diarrhea has typically ranked among the top causes of young child mortality and has been more deadly than measles and malaria (Bryce et al., 2005; Levine et al., 2012). Countries which are least developed often have the highest rates of childhood mortality; nearly three-quarters of diarrheal mortality occurs in 15 countries in sub-Saharan Africa and southern Asia (Boschi-Pinto et al., 2008; Levine et al., 2012). India and Bangladesh are among the top countries for diarrhea-related child mortality. Diarrhea-related causes are responsible for 9–13% of deaths for children under 5 in Bangladesh (Liu et al., 2011) and over 22% in India (Million Death Study Collaborators et al., 2010), resulting in hundreds of thousands of preventable deaths each year.

Even mild and moderate bouts of diarrhea can compromise child health and development, leading to conditions such as undernutrition (Humphrey, 2009; Dangour et al., 2013) and poor school attendance (Engle et al., 2011; Prüss-Ustün et al., 2014). Diarrhea is caused by viruses, bacteria, and parasites (Guerrant et al., 1990), disproportionately affecting people in developing countries due to issues with water, sanitation, and hygiene (WASH) (Montgomery and Elimelech, 2007). Recent statistics have shown that in India and Bangladesh, around 8% of children under 5 have had diarrhea in the last 14 days (Nasrin et al., 2013).

Good hygiene practices can significantly reduce rates of diarrhea (Fewtrell et al., 2005; Bartram and Cairncross, 2010), making sanitation and health behaviors a critical area for intervention. Worldwide, an estimated 2.4 billion people lack access to improved sanitation with 946 million people practicing open defecation (UNICEF and WHO, 2015). Studies conducted in India have shown that most people continue to defecate outdoors even with adequate access to latrines and toilets (Barnard et al., 2013). Increasing use of latrines is an effective strategy for improving sanitation and hygiene (Garn et al., 2017), thereby reducing rates of illness and death. Sanitation has been found to lower rates of diarrheal-related disease by 35%, showing it to be more effective at reducing diarrhea than improvements in water quality or quantity (Esrey et al., 1991).

There are many challenges to improving sanitation and hygiene. There have been several large-scale programs in South Asia to increase the number of available latrines, often involving subsidizing infrastructural developments related to latrine availability (Hueso and Bell, 2013). However, evaluations of these programs have shown that even with available infrastructure use of latrines is often low (Sanan and Moulik, 2007; Devine, 2009). This pilot study focuses on improving knowledge, attitudes, and behavior surrounding latrine use, shoe wearing, and handwashing. A previous study in India focusing on adult defecation habits where latrine use had increased after educational interventions found that children were still more likely to be found defecating in the open (Murthy et al., 1990) showing the need to focus on working with children. WASH educational interventions have been effective in improving hygiene behaviors (Garn et al., 2017); therefore, an intervention focused directly on educating children is a strength of this work. Additionally, households with low socioeconomic status, low levels of education, and low media exposure are less likely to use sanitary latrines (Akter et al., 2014). This is most common in rural areas (Yusuf and Zakir Hussain, 1990), which are the target of this intervention.

Cultural attitudes toward the concept of using a latrine for defecation have been shown to contribute to resistance to adopt latrine use (Bonu and Kim, 2009). Culturally appropriate health behavior change interventions are necessary to increase latrine use. Interventions that improve hygiene and offer sufficient footwear can decrease the number of childhood cases of diarrheal disease, parasitic infections, and anemia (Huda et al., 2012). Access to clean water, basic sanitation, nutrition, and immunization affect child mortality rates (Ghosh, 2012). The 2030 Sustainable Development Goals include “access to adequate and equitable sanitation and hygiene for all,” “stopping open defecation,” and “ending preventable deaths of newborns and children under 5 years of age” (United Nations, 2016).

Communication is crucial in encouraging individuals to adopt healthy behaviors (Sood et al., 2014). One method that has proven successful in shaping behavior is the use of media (Karanesheva, 2015). Researchers have documented how age-appropriate, specific, and intentional media content—rather than the medium itself—can influence education and health (Anderson et al., 2001; Wright et al., 2001; Mares and Pan, 2013; Sood et al., 2014). Findings are especially true for communication interventions in developing countries, with community-based campaigns being the most common practice (Sood et al., 2014). Communitybased research often takes places in disadvantaged communities where individuals are organized to participate with public health workers and researchers as partners in finding ways to improve health (Blumenthal et al., 2013). It is important for researchers to know the assumptions that are brought into research and to adopt a post-positivist view to better understand the implications of acting on what is thought to beneficial for another community (Ryan, 2006). In this work, there was continual communication with stakeholders in the community as well as with in-country researchers and local WASH experts to try to ensure that the intervention content and implementation were created with input from the communities it was targeted to serve.

Mass media, such as television, has been useful in disseminating health messages in a cost-effective way to individuals, even for those living in rural areas (Karanesheva, 2015). Entertainment education initiatives to promote health and change health behaviors have been implemented throughout the world (Brown, 2012). One example of this is *Sesame Street*, a children’s educational television program produced by Sesame Workshop, which has been a major producer of educational initiatives targeting children (Cole et al., 2008). *Sesame Street* has been on air for 47 years and is shown in over 150 countries around the world (Sesame Workshop, n.d.). In over 30 countries, locally produced coproductions of *Sesame Street* address the educational needs of children in specific countries or regions. A study in Bangladesh showed that parents and caregivers regarded *Sisimpur* (the Bangladeshi coproduction) as an important educational resource for both their children and themselves, and it was a well-known information source for children’s healthy practices (Kibria and Jain, 2009). A meta-analysis examining the effects of watching *Sesame Street* coproductions in low- and middle-income countries found an effect size of 0.29 (Mares and Pan, 2013), which was comparable to other early childhood interventions, with the distinction of scale: *Sesame Street* regularly reaches over 156 million children worldwide (Sesame Workshop, n.d.). Due to its popularity and high appeal, *Sesame Street* is an effective channel for reaching many children and conveying important messages. In addition to mass media, Sesame Workshop works with partners in many countries to implement community- and school-based programs that use multiple channels and resources (audio-visual, print, digital) to engage children.

*Sesame Street* has had a presence in Bangladesh and India for about 10 years, in the form of locally produced *Sesame Street* coproductions *Sisimpur* and *Galli Galli Sim Sim*, created by in-country educators and producers to meet the educational needs of young children in each country. Sesame Workshop has established offices in both countries (headed and staffed by Bangladeshi and Indian counterparts) to create locally relevant educational content for mass media platforms, schools, and community settings.

The Bill & Melinda Gates Foundation funded Sesame Workshop for “*Cleaner, Healthier, Happier*” because they saw the need for WASH behavior change communication programs targeted at young children and saw Sesame Workshop’s in-country presence in Bangladesh and India and the success of *Sisimpur* and *Galli Galli Sim Sim* as strengths. The teams from Sesame Workshop Bangladesh and Sesame Workshop India developed the “*Cleaner, Healthier, Happier*” content, co-created Raya, and adapted its implementation in high-need communities. Raya arose from a unique set of needs: there was a need for a strong young girl character at the heart of the project as WASH issues disproportionately affect girls and young women (Sommer et al., 2015), yet none of the existing options—using a US Muppet character, using a Bangladeshi Muppet character (i.e., familiar in Bangladesh but unfamiliar to other audiences), or using an Indian Muppet character—were ideal; each met with resistance in some way. There were also ambitions for “*Cleaner, Healthier, Happier*” to be a global behavior change communication program, with a character who will be readily identifiable with WASH issues, who is relatable across geographies, and could serve as a child-friendly messenger for WASH. Thus, a new character had to be created. Raya was designed and created with full and equal input from in-country teams, as was all content that featured her. In-country teams also tested Raya with children during the production process to ensure that she resonated locally. “*Cleaner, Happier, Healthier*” was created with local relevance and meaningful impact in mind at every stage of the project.

This paper describes research examining the educational impact of a small-scale pilot of the “*Cleaner, Happier, Healthier*” intervention in Sylhet, Bangladesh and Kolkata, India. The overall purpose of the intervention was to promote WASH knowledge, attitudes, and behaviors among some of the most vulnerable children and families of these countries using a multi-media health communication approach. The country teams created interventions that utilized two different approaches to WASH messaging: one that appealed to personal motivations for behaviors and one that appealed to social motivations. The study examined both the overall impact of the interventions, as well as the relative efficacy of these two approaches. Thus, the guiding research questions were “Does exposure to the ‘*Cleaner, Happier, Healthier’* intervention have a positive impact on children’s WASH knowledge, attitudes, and behaviors?” and “Does a personal-based or a social-based messaging approach have greater impact on changing WASH knowledge, attitudes, and behaviors?” We expected that children in the intervention groups would have greater positive change in WASH knowledge, attitudes, and behaviors than children in the comparison group; however, it was unknown as to which message approach—personal versus social—would be more effective.

Including two forms of messaging was a novel approach as there was no existing literature on which approach may be more effective, or if there would be differences in effectiveness based on the message approach, but we thought that the patterns of findings may differ depending on the country, the behavior/outcome, and the child’s age. The targeted WASH behaviors— using a latrine, washing one’s hands, and wearing sandals—are all individual behaviors and would generally be conceptualized as such (e.g., the girl washes her hands, the boy uses the latrine). Yet the Principal Investigator and the Sesame Workshop research team also recognized that hygiene is a public health practice and personal behaviors foster community health. Therefore, personal and social health messaging approaches were used in the intervention to allow for a comparison of these different types of messaging. This concept was reviewed and approved by the project manager in each country.

## THE INTERVENTION

In 2013, the Bill & Melinda Gates Foundation awarded funding to Sesame Workshop to develop a public health intervention in Bangladesh, India, and Nigeria. These locations were chosen due to great need in these areas. Due to the similarity in implementation, this paper focuses on the programs in Bangladesh and India. We acknowledge that while these countries share many cultural similarities, some WASH behavioral differences exist in these two locations which may contribute to differences in the findings regarding the intervention effects for each country. In addition to great need for WASH interventions in these countries, practically it was important to be able to create one intervention that could benefit people in multiple areas. The intervention was developed for delivery in both locations and this manuscript describes the implementation and results from both countries.

The purpose of the intervention was to increase positive WASH practices among children and their families in these countries. Program components included various forms of media and in-person activities. A new Muppet^®^, a teal-colored girl puppet named “Raya,” was developed and introduced; this character advocated for health behaviors including using a safe latrine, wearing sandals, and handwashing. Raya was developed to be an appealing and relatable character for children in both Bangladesh and India. Raya is an energetic 6-year-old with long dark braided hair and wears a yellow embroidered kurta. She talks about latrines and defecation in ways in which children from Bangladesh and India can identify.

Although there are some differences in the geographic areas chosen to implement this pilot program, there are also many similarities. While most Sylhet Bangladeshis are Muslim and most Kolkata Indians are Hindu, they share many historical and cultural characteristics. Food and diets resemble each other, with an emphasis on fish, vegetables, lentils, and rice. While both maintain traditional gender and family roles, there are challenges in terms of girls’ education and child labor. Although different dialects are spoken, Bengali is the dominant language in both locations. Many of the same writers, artists, and musicians are celebrated by both Indians and Bangladeshis. Finally, people of this region share physical traits in terms of skin, hair, and eye color, as well as clothing styles.

While this was a short-term pilot intervention, care was taken to involve the communities in the design and implementation of the “*Cleaner, Healthier, Happier*” program. In-country teams, in consultation with local WASH experts, identified educational objectives and target communities. Project managers in each country were both from local universities and had background in early child development as well as skill in local language dialects. Prior to the implementation of the study, the Principal Investigator traveled to both India and Bangladesh for 1 week training sessions to discuss the ethics of data collection among vulnerable populations and reviewing and revising the survey instruments. Needs assessments were conducted in Kolkata, India and Sylhet, Bangladesh with community members to understand their knowledge, attitudes, and behaviors around hygiene and sanitation practices. This information was key to addressing gaps in communication and formed the foundation for content development and implementation strategy, aiding in refining the education objectives. Formative research was also conducted with children in these areas during the development of content, ensuring the materials were relatable to the target audience. Community members were also engaged during the implementation phase of this intervention. Community volunteer workers with a background in delivering content to young children were chosen by an NGO partner in each country. These volunteers aided in delivering the intervention material to children and families and created the timeline of activities, as they were most knowledgeable about the area and what could be completed in the desired time frame with the targeted audience.

Previous work on WASH behaviors has not focused on examining the impact of intervention messaging based on whether it is viewed as a personal or social benefit to participants. That is why this study developed the “me” (personal) and “we” (social) approach to health messaging. This dichotomy was selected for study, as hygiene behaviors involve both aspects. Using the latrine and handwashing is an individual behavior. Around the globe, the norm is for people to urinate and defecate by themselves. A boy puts on and wears his own sandals, a girl washes her own hands. That said, hygiene is a public health practice. Personal behaviors foster and affect the health of a community.

The pilot program explored different approaches in health messaging (see **Table 1**). One approach involved framing hygiene and sanitation as a personal behavior (the “me” intervention); participants were told that engaging in these behaviors would benefit the individual, making him or her healthier. The other approach considered hygiene and sanitation as a social endeavor (the “we” intervention). Children who received this messaging were encouraged to use improved behaviors to improve the health of their peer group and the community. These different approaches were overt in the program’s messaging. For example, individual motivation text using the “me” approach may read “*I* wear sandals every time *I* go to the latrine; it keeps *me* healthy and clean” focusing on the benefits for the singular participant, while the social motivation or “we” approach text reads “*we* wear sandals every time *we* go to the latrine; it keeps *us* healthy and clean” focusing on the impact of the action on multiple members of the community. All of the print materials’ text and images (in Bangladesh), the organization of the activities (in India), the facilitator training, and the facilitation of the program activities were either “me” or “we” focused.

**TABLE 1:** Components of *Cleaner, Healthier, Happier* intervention.

Project component	Variation description	example
Messaging	Participants in the individual motivation were encouraged to engage in sanitation and hygiene behaviors to maintain their own health Participants in the social motivation were encouraged to engage in sanitation and hygiene behaviors to contribute to the health and cleanliness of their community	It is important to always wash my hands after using the toilet because it keeps me healthy and germ-free! It is important for everyone to always wash our hands after using the toilet because it keeps our community healthy and germ-free!
Print material—text	The print material focuses on individual motivation depicted a single child practicing and participating specific sanitation and hygiene behaviors that would keep her healthier. The print text was constructed to encourage and support the individual child as responsible for contributing to her own health On the other hand, the print material text focuses on social motivation by addressing the child, her friends, family members, and her community in terms of the need for everyone practice and participate in specific sanitation and hygiene behaviors that would keep them healthier. The print text was constructed to encourage and contribute to the health and cleanliness of their community	For an individual motivation example, the text reads, “I wear sandals every time I go to latrine; it keeps me healthy and clean.” For a social motivation example, the text reads, “We wear sandals every time we go to latrine; it keeps us healthy and clean.”
Print material—images	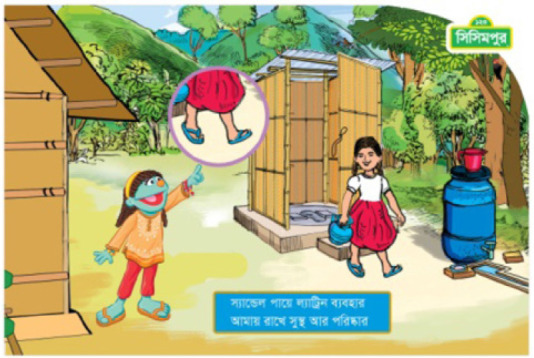 Image for individual motivation shows a girl coming out from the latrine wearing sandals and holding a water pot. Raya points to the girl’s feet to reinforce the personal behavior. 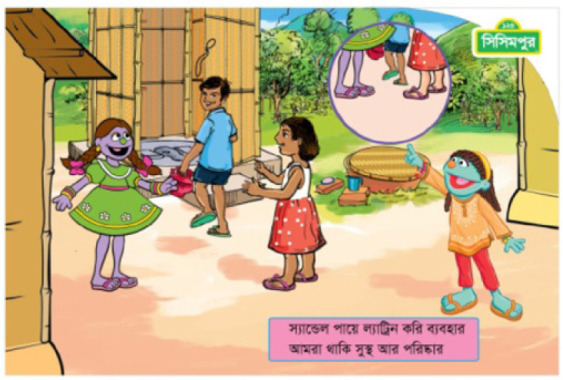 Image on the social motivation shows a girl, a boy, and Tuktuki, another Muppet character, wearing sandals as the boy approaches the latrine. Raya points to everyone’s feet to reinforce the group behavior.	*Please see the images to the left from Bangladesh as examples*
Facilitator training	Facilitators training for personal motivation and social motivation were conducted separately to separate groups of facilitators. Separate instructors and training guides were used to train each messaging group. In the personal motivation facilitator group, training focused on personal/individual behaviors related to sanitation and hygiene. In the social motivation facilitator group, training focused on family and community behaviors	Individual motivation training focused on print materials depicting one child and on personal messaging. Examples and questions were centered on “me” in relation to sanitation and hygiene behaviors. Social motivation training focused on print materials depicting groups and on social messaging. Examples and questions were centered on “us” in relation to the desired behaviors
Facilitation	During the intervention, facilitation that drew on individual motivation focused on each child’s behaviors related to sanitation and hygiene. Examples were presented and discussed that centered on how a child’s own behaviors can support her health and cleanliness During the social motivation activities, conversations and examples were centered on family, friends, and the community. The facilitator focused on how everyone’s behaviors can support the health and cleanliness of a community	During the personal motivation intervention, the facilitator focused on an individual practicing sanitation and hygiene behaviors. The facilitator might ask, “What is Raya doing after using latrine? Do you do the same?” During the social motivation intervention, the facilitator focused on groups, families, and the community practicing sanitation and hygiene behaviors. The facilitator might ask, “What are Raya and Elmo wearing? Why do we all need to wear sandals to the latrine? What do we all have to do after using latrine in order stay healthy and clean?”

In both countries, the study delivered the different approaches in two very similar communities, with one community in each country receiving the “me” intervention and another the “we” intervention. Additionally, data were collected in a comparison community in each country, where no intervention was done. The population density, program design, and scope of the pilot program prevented a design where we could randomize participants into one of the three groups. As a result, the design here involved a non-randomized study, where we compared those receiving the intervention to the comparison group. We also evaluated differences between those in the “me” and “we” interventions communities.

The pilot intervention took place over a period of 3 months in 2014 (in Bangladesh) and 2014/2015 (in India). The programs in both countries consisted of community-targeted mobile viewings where participants watched videos created for the project, followed by simple activities led by a facilitator such as small group games, stories to reinforce WASH messages, and activity sheets and child-targeted activities or workshops led by trained facilitators. Participants in Bangladesh had approximately 12 exposures to the project’s content and in India, participants had approximately 24 exposures. (The 24 exposures in India were a result of implementing the mobile community viewings and workshops separately; whereas in Bangladesh, the activities were implemented closely together.) Exposures happened in community spaces or at school facilities (activities were conducted in school facilities but happened after school hours and were open to everyone in the community). Young children were registered to participate in the program and were encouraged and reminded to attend. Program content and activities were specifically targeted at children, but parents and community members were also encouraged to attend.

The intervention materials and activities were delivered by NGO partners in each country. Various media materials used included video (with WASH-relevant Muppet segments/inserts, live action films, and Sesame Workshop library content) and print resources (floor games, flip charts, books, and activity sheets). India had additional digital games developed for this project because the program team in India had previous experience implementing such activities and saw them as an important, engaging, and motivating component of the project. In Bangladesh, the intervention happened in very remote rural tea gardens without a reliable electricity supply; a tablet-based element would not have been feasible. However, even though the medium of delivery may be different, care was taken to ensure that the WASH curricular messages were repeated and reinforced to the same extent in both countries with a set curriculum delivering the same health messaging and similar amount of exposure to the intervention materials for participants in each country. Lessons and themes reinforced through the produced materials were the same in both countries and included using the latrine, wearing sandals in general and to the latrine, washing hands, washing hands with soap, and safe water transportation and storage. Children were exposed to all content and activities in their community or through schools in Bangladesh, whereas adult caregivers were largely exposed only to the mobile community viewings, which were accessible to all members of the community.

### Methodology

The Principal Investigator, in consultation with Sesame Workshop, created the research instruments and protocols. Great care was given to the research instruments so that questions were developmentally and culturally appropriate. This research began with a common survey in English. This was presented to the research team in each country for translation. It was then reviewed by “gate-keepers” in the chosen communities. Edits were discussed and incorporated. Because Sesame Workshop is an educational media organization, this program focused on messaging and behavior change communication, not the provision of infrastructure. Within the WASH development community, there is a strong acknowledgment of the need for educational messages and to start early in inculcating WASH practices. Questions were refined after some pilot-testing and reviewed by child development experts in the United States, Bangladesh, and India. The research Project Director in each country convened a team of a dozen in-country researchers, all of whom had experience working with young children prior to this study and led training sessions, which were intensive and in person.

In-country researcher teams carefully chose community-based workers to take the messages of the project to the children and community members. These community workers were either referred by other community workers or had previously worked on projects led by NGO partners and were all from local communities. Once selected, the volunteer facilitators received multi-day training to implement the “*Cleaner, Healthier, Happier*” program with either a focus on social motives (the “we” group) or individual motives (the “me” group). The training sessions were pilot tested by participants associated with the project prior to being conducted with the facilitators. Training focused on acquainting facilitators with the in-country productions of *Sesame Street* and the “*Cleaner, Healthier, Happier*” campaign, as well as teaching facilitation and monitoring skills, ensuring understanding of all campaign materials, and practicing mock sessions with workshop activities. The sessions focused on specific skills so that the data collection with the children and parents would be ethical, reliable, and valid.

In-country Project Directors each recruited teams of a dozen researchers. The Project Directors led training sessions, which were intensive and in person. Following the completion of their training, researchers conducted one-on-one, in-person interviews with each participant, in his or her preferred language. All interviews were conducted by researchers who were familiar not only with the communities’ language but also with the local dialect. It was critical that the children understand the words and even accents of the researchers. Surveys done before and after the intervention took around 40 min for the parent/guardian and 30 min for the child participants. In keeping with recommendations for interviewing this age group, children’s answers were mostly multiple choice or close-ended (Borgers et al., 2000). Interview sessions with children were conducted by researchers who had previous experience with this age group and the experience was kept engaging and separated into short sections to maximize the reliability of children’s responses (Vaillancourt, 1973). Responses were recorded on a paper and pencil data sheet. While the intervention activities were delivered in community spaces or school facilities after school hours, in order to reach as many participants as possible for the parent–child interviews, the majority of interviews were conducted just outside of the participant’s home. Researchers tried to conduct the child interviews within sight but not ear-shot of the parents to avoid parents prompting responses from their children.

Researchers explained the study, and informed parental consent and child assent were obtained prior to data collection. Because of low literacy in the selected research areas, an oral consent procedure was used for both adults and children. Quality control of data collection was enforced by the in-country research teams. In Bangladesh one Project Coordinator and two Assistant Project Coordinators oversaw eight researchers. In India, a field manager was responsible for every five researchers, and at least 5% of the research sessions were re-visited in person by the manager. Double data entry with a validation check occurred. Finally, a check was done by the UMD team, confirming that evaluation participant children were, in fact, part of the intervention groups. The School of Public Health, University of Maryland’s Institutional Review Board reviewed and approved the protocols and instruments.

In Bangladesh, the intervention and study occurred in three locations (Bhararura, Bhurbhuria, and Satgaon), all in the Moulvibazar District of Bangladesh’s Sylhet Division. Sylhet Division is in northeast Bangladesh, along the River Surma, and is bordered by India on the north and east. Sylhet is home to a large number of indigenous tribal communities, who are considered to be ethnic minorities in Bangladesh. The landscape is distinctive with sub-tropical hills; tea estates are in abundance in this area. The area has over 150 tea estates, and nearly 300,000 employers work these estates. Many people from Sylhet work intensively, with few tools, and for a meager wage. Tea estate laborers live in densely populated slums, which lack proper sanitation. There is no formal plumbing or piped water in this area. For most people, the main source of drinking water is ground or surface water. Wells, ponds, rivers, and/or springs are used for washing and cleaning clothes, bathing, and even for washing utensils. The majority of households and public buildings (like schools) lack a water-sealed latrine. People usually defecate in the tea gardens or near springs.

The Sylhet region has poor health indicators; people of the tea estates suffer from a variety of illness, including headaches, skin diseases, hookworm, fever, malaria, cough and cold, acute respiratory infection among the children, gastric/ulcer, blood pressure, toothache, diarrhea, jaundice, and dysentery. According to the 2014 Bangladesh Demographic and Health Survey [National Institute of Population Research and Training (NIPORT) et al., 2016], Sylhet has the highest under-5 mortality rate among all the divisions. Additionally, around 6% of children in the Sylhet region had diarrhea in the previous 2 weeks [National Institute of Population Research and Training (NIPORT) et al., 2016]. The three communities chosen for this pilot study, Bhararura, Bhurbhuria, and Satgaon, greatly resemble each other in terms of landscape and demographics. Baseline data collection occurred in March and April of 2014 and post-intervention data collection in July and August of 2014. In each community, schools provided rosters listing community children between the ages of 3 and 8 years. From these lists, 80 children were randomly selected per community. For each selected child, parents/guardians were identified and approached. In total, data were collected from 240 parent/child pairs in Bangladesh at baseline and follow-up.

In India, the “*Cleaner, Healthier, Happier”* intervention occurred in two slums within Kolkata. Kolkata (formerly Calcutta) is a modern Indian city, and capital of the state of West Bengal. Kolkata sits on the Hooghly River; the city and suburbs is home to more than 14 million people. Despite being a center for commerce, culture, and education, poverty is a profound problem in Kolkata. According to a recent source, almost a fourth of the population lives on less than 27 rupees a day (around 0.45 US dollars). A third of Kolkata’s population lives in its slums and another 70,000 are completely homeless. Poor living conditions are of concern in Kolkata’s slums. Crowding is evident, with more than one in five households having at least seven persons per sleeping room. There is poor ventilation in these small homes, and less than 20% of the households have windows (Gupta et al., 2009). Around 85% of the households have access to piped drinking water but only 24% use an improved toilet facility, such as a ventilated latrine that is not shared with other families in the community (Gupta et al., 2009). A third of the homes in the Kolkata slums have a separate kitchen; 13.5% cook meals right outside of the home. Around half (52.4%) of Kolkata slum residents use kerosene or coal as their cooking fuel, and another 35.4% have electricity.

Communities who received the intervention were chosen from different areas: one in the Jorasanko area (Ward 39, with some participants from Ward 41 and Ward 44), the other in the Narkeldanga area (Ward 28). Children in the comparison group were also drawn from the Joransanko area, but from Ward 37. Wards were separated by several kilometers, and the non-profit organization Children’s International (which implemented the program) assisted with determining where work could occur without the probability of contamination across Wards. These slum areas and Wards are very similar in terms of households and demographics. While the definition of slum is being reconsidered in India (Patel et al., 2014), it is understood that people living in this area lacked access to water, sanitation, space, and durable house structure. Baseline data collection occurred in November 2014; post-intervention data collection followed in February 2015. Sesame Workshop India provided the research team with a roster of all children registered for the intervention. Then, the research team randomly selected children for the evaluation study. For each selected child, parents and guardians were identified and approached. In India, data were collected from 258 parent/child pairs at baseline and 223 at follow-up.

#### Message Focus

In Bangladesh, children from Bhurbhuria received the “me” approach intervention, where messages and activities were framed as having an individual motivation. Children from Bhararura had the “we” approach, where intervention messages and activities addressed hygiene and sanitation as community and social endeavors. Participants from Satgaon served as a comparison group and did not receive hygiene and sanitation lessons.

In India, participating children from the Jorasanko area received the “me” approach and children from Narkeldanga area received the “we” approach. Children from a separate Jorasanko Ward were the comparison group and did not have hygiene nor sanitation lessons.

### Measures and analyses

#### Sanitation and Hygiene Outcomes

The study’s main outcomes focused on knowledge, attitudes, and behaviors related to using a latrine, wearing sandals, and handwashing. The research instruments and protocols were developed by the Principal Investigator in consultation with Sesame Workshop. Questions were refined after pilot-testing and reviewed by child development experts in the United States including the Principal Investigator and Sesame Workshop staff, and in India and Bangladesh, questions were reviewed by the in-country research team Project Directors. Semi-structured questionnaires were developed by the research team at the University of Maryland, and then reviewed by the local partners (The Bangladesh Center for Communication Programs and Policy Innovations in India) for developmental and cultural appropriateness.

#### Knowledge

Adults were asked knowledge questions at baseline and after the intervention (eight questions in Bangladesh, seven in India). These questions included: “Why should children use latrines/toilets?” “Why should children wear slippers/sandals when they go to the toilet?” “Why should children wash their hands, why should one pour water after using the toilet?” “What should you do with children’s excreta and why?” “Why is it important to have clean latrines?” and “What water is safe to drink?” An additional question, “What is a tippy tap?” was only asked in Bangladesh. Correct responses were scored as a “1” while incorrect and “don’t know” responses were coded as a “0.”

For child participants, a knowledge score was created summing positive responses to several statements (six in Bangladesh, seven in India), both at baseline and after the intervention. These statements included: “Using the latrine can help keep you healthy,” “it is important to wash your hands,” “not washing your hands can get you sick,” “it is important to wear shoes,” “wearing shoes can keep you healthy,” and “wearing shoes can keep you safe.” In India an additional question “What should you use to wash your hands after using the latrine?” was also asked. Only responses where the child said both soap and water were marked as correct. In Bangladesh at baseline, the alpha was 0.51 and at post-intervention, it was 0.49. In India at baseline the alpha was 0.55 and at post-intervention it was 0.39.

#### Attitudes

Parents and children reacted to several statements, before and after the intervention, to create a hygiene attitudes scale. In Bangladesh and India, parents reacted to the following eight items at both assessments:

It is not necessary to deposit babies’ excreta in latrines.

Going in the open prevents germs from spreading.^1^

Children younger than 10 years old do not need to wear sandals to the latrine (see text footnote 1).

It is healthy to use latrines.

It is fine to drink water from an earthen well or pond (see text footnote 1).

Water containers must always be covered.

It is the adults’ responsibility to collect water in a safe way. It is important to use latrines.

In Bangladesh, an additional six items were asked of parents, including:

Diarrhea can be prevented.

Unclean latrines can lead to disease.

Adults should begin to prepare children to use a latrine at age 3 years.

Soap prevents diseases.

Adults should make sure that latrines are clean for children. Handwashing needs to happen every time a person uses the latrine.

During baseline, all items were scored on a 5-point Likert scale with higher scores representing healthier attitudes. During post-intervention all items were scored on a 3-point Likert scale with higher scores representing healthier attitudes. For analysis, the original baseline 5-point scale was recoded into a 3-point scale thereby giving the Adult’s General attitude scale a range of 8–24 with higher scores indicating healthier attitudes. In Bangladesh, the Cronbach’s alpha was 0.57 at baseline and 0.59 at post-intervention (14 items). In India, parents reflected on eight statements at baseline, and six at the post-intervention assessment (two items which had zero variance were removed from the follow-up survey.) In India, the Cronbach’s alpha was 0.89 at baseline (8 items) and 0.23 at post-intervention (6 items).

To assess attitudes about hygiene, children considered on a four point scale how much they agreed or disagreed with the following six statements. They were:

The latrine is the only good place to defecate.

If you have to go, it is fine to go in the field/bushes (see text footnote 1).

Everyone should wash his or her hands after going to defecate. It does not matter if you wash after using the latrine (see text footnote 1).

Everyone should wear shoes when he or she has to defecate. Drinking clean water will keep you healthy.

Again, internal consistency for this scale was low. The Cronbach’s alpha for this scale was 0.31 at baseline, 0.41 at post-intervention in Bangladesh and 0.49 at baseline and 0.51 at post-intervention in India. Low alphas for attitudes reflect the possibility that children may feel differently about certain concepts and activities; for example, a child might be care considerably about drinking clean water but be less concerned about handwashing. Low alphas were expected here due to combining questions concerning so many different targeted attitudes and behaviors. These scales were developed to determine overall knowledge and attitudes concerning the targeted WASH behaviors. To compare variables across the countries, samples of parents and children, and from baseline to post, the knowledge and attitude scales were standardized. Baseline scores and post-intervention scores were pooled and we created standardized *z* scores. As a result, we were able to calculate for each individual a standardized change score. A negative standardized change score would suggest an average decrease in knowledge or attitudes for the group, while a positive standardized change score would indicate an increase, on average, for the group.

#### Behaviors

Parents and children were asked about various sanitation and hygiene behaviors. Researchers asked about *the last time* (i.e., such as the last time the child defecated) and the *frequency* that a child typically engaged in a behavior. Among the variables considered were whether the child used a latrine at home and at school (only asked of the parent), wearing sandals outside the house, wearing sandals to the latrine, handwashing after defecating, and handwashing with a cleanser/soap. Change scores were calculated for each behavior as post-intervention minus baseline scores. Higher frequency of desired behaviors resulted in higher change scores. For these behaviors we were able to create a three-point scale for each child. Here, a 0 would mean no behavior change, a +1 would indicate a shift from not engaging to engaging in a positive behavior, and a −1 would imply that the child had engaged in a positive behavior at the start but not after the intervention.

#### Demographics

From adults, demographic data were collected including children’s age and gender, number of people in the household, main language spoken in the home, highest level of education achieved by any adult member of the household and access to household resources (see Tables 2 and 3).

**TABLE 2:** Basic information about the samples from Bangladesh (*N* = 240) and India (*N* = 258).

Variable	Bangladesh				India			
	Total	Me	We	comparison	Total	Me	We	comparison
	*N* = 240	*N* = 80	*N* = 80	*N* = 80	*N* = 258	*N* = 83	*N* = 86	*N* = 89
Gender, *N* (%)								
Girls	111 (46.3)	30 (37.5)	35 (43.8)	46 (57.5)	131 (50.8)	39 (47.0)	47 (54.7)	45 (50.6)
Boys	129 (53.8)	50 (62.5)	45 (56.3)	34 (42.5)	127 (49.2)	44 (53.0)	39 (45.3)	44 (49.4)
Age (in years), Mean (SD)	6.2 (0.90)	6.3 (0.9)	6.1 (0.9)	6.3 (0.9)	5.63 (1.7)	5.56 (1.7)	5.87 (1.8)	5.48 (1.6)
**Highest education for any household adult, N (%)**								
Non-literate	1 (0.4)	0 (0.0)	1 (1.3)	0 (0.0)	40 (15.5)	11 (13.3)	8 (9.3)	21 (23.6)
Literate, no formal schooling	7 (2.9)	3 (3.8)	2 (2.5)	2 (2.5)	33 (12.8)	15 (18.1)	10 (11.6)	8 (9.0)
Completed primary school (or less)	97 (40.4)	39 (48.8)	30 (37.5)	28 (35.0)	92 (35.7)	32 (38.6)	28 (32.6)	32 (40.0)
Completed secondary school	113 (47.1)	44 (41.3)	37 (46.3)	44 (53.8)	57 (22.1)	12 (14.5)	26 (30.2)	19 (21.3)
Beyond high school	22 (9.1)	5 (6.3)	10 (12.5)	7 (8.8)	21 (12.1)	9 (10.8)	13 (15.2)	9 (10.1)

**TABLE 3:** Household resources of the sample, as described by parents before the intervention.

	Bangladesh				India			
	Total	Me	We	comparison	Total	Me	We	comparison
	*N* = 240	*N* = 80	*N* = 80	*N* = 80	*N* = 258	*N* = 83	*N* = 86	*N* = 89
Ownership of household items, *N* (%)								
Electricity	143 (59.6)	42 (52.5)	52 (65.0)	49 (61.3)	253 (98.1)	80 (96.4)	84 (97.7)	89 (100.0)
Radio	3 (1.3)	1 (1.3)	1 (1.3)	1 (1.3)	22 (8.5)	4 (4.8)	10 (11.6)	8 (9.0)
Black and white TV	25 (10.4)	6 (7.5)	10 (12.5)	9 (11.3)	13 (5.0)	7 (8.4)	3 (3.5)	3 (3.4)
Color TV	63 (26.3)	18 (22.5)	25 (31.3)	20 (25.0)	209 (81.0)	59 (71.1)	77 (89.5)	73 (82.0)
VCR/DVD player	16 (6.7)	5 (6.3)	5 (6.3)	5 (7.5)	24 (9.3)	9 (10.8)	5 (5.8)	10 (11.2)
Motorcycle	2 (0.8)	0 (0.0)	1 (1.3)	1 (1.3)	12 (4.7)	2 (2.4)	5 (5.8)	5 (5.6)
Car	0 (0.0)	0 (0.0)	0 (0.0)	0 (0.0)	1 (0.4)	0 (0.0)	1 (1.2)	0 (0.0)
Clothes washing machine	0 (0.0)	0 (0.0)	0 (0.0)	0 (0.0)	4 (1.6)	0 (0.0)	1 (1.2)	3 (3.4)
Refrigerator	1 (0.4)	0 (0.0)	1 (1.3)	0 (0.0)	63 (24.4)	18 (21.7)	24 (27.9)	21 (23.6)
Fixed line telephone	4 (1.7)	1 (1.3)	3 (3.8)	0 (0.0)	3 (1.2)	0 (0.0)	3 (3.5)	0 (0.0)
Mobile phone	140 (58.3)	46 (57.5)	46 (57.5)	48 (60.0)	242 (93.8)	76 (91.6)	82 (95.3)	84 (94.4)
Computer	1 (0.4)	0 (0.0)	0 (0.0)	1 (1.3)	5 (1.9)	0 (0.0)	3 (3.5)	2 (2.2)
Access to the internet	0 (0.0)	0 (0.0)	0 (0.0)	0 (0.0)	50 (19.4)	16 (19.3)	17 (19.8)	17 (19.1)
**Drinking water**								
Piped into household	0 (0.0)	0 (0.0)	0 (0.0)	0 (0.0)	35 (13.6)	16 (19.3)	8 (9.3)	11 (12.4)
Piped into compound	1 (0.4)	1 (1.3)	0 (0.0)	0 (0.0)	211 (81.8)	67 (80.7)	74 (86.0)	70 (78.7)
Tube well	238 (99.2)	80 (100.0)	80 (100.0)	78 (97.5)	1 (0.4)	0 (0.0)	1 (1.2)	0 (0.0)
Other	10 (4.2)	6 (7.5)	1 (1.3)	3 (3.8)	11 (4.3)	0 (0.0)	3 (3.5)	8 (9.0)
**Toilet facility used by child**								
No facility/street/outside/bush/field	116 (48.3)	49 (61.3)	31 (38.8)	36 (45.0)	10 (3.9)	3 (3.6)	5 (5.8)	2 (2.2)
Traditional pit toilet	98 (40.8)	24 (30.0)	38 (47.5)	36 (45.0)	122 (47.3)	56 (67.5)	29 (33.7)	37 (41.6)
Ventilated improved pit latrine	26 (10.8)	7 (8.8)	11 (13.8)	8 (10.0)	114 (44.2)	16 (19.3)	50 (58.1)	48 (53.9)
Flush toilet	–	–	–	–	8 (3.1)	5 (6.0)	1 (1.2)	2 (2.2)
Other	–	–	–	–	4 (1.6)	3 (3.6)	1 (1.2)	0 (0.0)

*Not all totals add to total number of participants (especially for water used). Some participants get water from multiple sources*.

#### Analyses

The interventions took place at the community level (i.e., in both countries, two communities received one of the approaches and one served as a comparison group). First, the researchers compared the communities for differences in demographics and household variables (see Tables 2 and 3). Then, researchers examined outcome variables, examining trends and patterns for each. Next, the researchers developed composite scores and scales for many of the constructs.

For analyses of knowledge and attitudes, standardized scores were developed and the study considered change (see Tables 4 and 5). Change scores were created by subtracting the baseline score from the post-intervention score. This allowed for comparisons to be made across the knowledge and attitude variables as well as across groups. For categorical variables, researchers considered the status of a given behavior (i.e., adopting a behavior, staying the same, giving up a behavior) or frequency of a behavior change (i.e., went from “some of the time” to “all of the time”) (see **Table 6**). Examining mean and variation change by group, we used ANOVA to test for statistical differences. Comparisons were made across the three groups (“me,” “we,” and comparison).

**TABLE 4:** Standardized change in knowledge and attitudes.

	Bangladesh				India			
standardized change score	Me	We	comparison	Sig.	Me	We	comparison	Sig.
Adult knowledge	1.40^a^	1.17^a^	-0.11^b^	*p* < 0.001	0.26	0.29	0.18	ns
Child knowledge	0.63	0.39	0.15	ns	0.33	0.39	0.44	ns
Adult general attitudes	0.34^a,b^	−0.11^a^	0.63^b^	*p* < 0.01	−0.88	−0.77	−0.84	ns
Child general attitudes	0.50^a^	0.38^a,b^	−0.04^b^	*p* < 0.05	0.27	0.35	0.28	ns

a,b *Different superscripts denote significant differences between groups at a p < 0.05 level for post hoc tests*.

**TABLE 6:** Change in behaviors.

Behavior change	Bangladesh				India			
	Me mean (sD)	We mean (sD)	comparison mean (sD)	Sig.	Me mean (sD)	We mean (sD)	comparison mean (sD)	Sig.
**Toileting**								
Improved toilet at home	0.25^a^ (0.54)	0.34^a^ (0.57)	0.01^b^ (0.25)	*p* < 0.001	0.60^a^ (0.70)	0.11^b^ (0.72)	0.21^b^ (0.53)	*p* < 0.001
Improved toilet at school	0.38^a^ (0.57)	0.40^a^ (0.0.63)	−0.01^b^ (0.82)	*p* < 0.05	0.16 (0.98)	0.32 (0.78)	0.15 (0.50)	ns
**Sandal/shoe wearing**								
“All the time” outside the house (parent response)	0.94 (1.20)	0.76 (0.98)	0.88 (1.01)	ns	0.45 (0.05)	0.51 (0.59)	0.57 (0.07)	ns
“All the time” when going to defecate (parent response)	1.00 (1.01)	0.77 (1.02)	0.81 (0.99)	ns	0.05^a^ (0.51)	0.35^b^ (0.58)	0.07^a^ (0.59)	*p* < 0.001
Wore last time at toilet (parent response)	0.68 (1.24)	0.38 (1.17)	0.34 (0.57)	ns	0.14 (0.71)	0.53 (1.42)	0.28 (1.04)	ns
**handwashing**								
Frequency immediately after defecation (parent response)	0.91^a^ (1.69)	0.81^a^ (1.57)	−0.15^b^ (1.20)	*p* < 0.01	0.08 (0.51)	0.26 (0.78)	0.10 (0.56)	ns
Soap frequency (parent response)	1.46^a^ (1.89)	1.04^a,b^ (1.81)	0.56^b^ (0.97)	*p* < 0.001	0.45^a,b^ (0.89)	0.51^a^ (1.06)	0.15^b^ (0.60)	*p* < 0.05
Soap frequency after defecation (parent response)	1.23^a^ (1.52)	0.95^a^ (1.43)	0.21^b^ (1.50)	*p* < 0.001	0.24 (1.18)	0.26 (0.91)	0.21 (0.61)	ns

a,b *Different superscripts denote significant difference between groups at a p < 0.05 level for post hoc tests*.

To test for statistical significance of the intervention (together and as separate approaches), we created linear regression models predicting change in knowledge, attitudes, and behaviors. Models controlled for child age, child gender, child baseline knowledge, and parent education, and used change scores for the knowledge, attitude, and behavior outcomes. The β values reported in the regression tables represent the change associated with the intervention (“me” and “we” combined) versus the comparison group for each target outcome. Full models for every outcome are available upon request from the corresponding author.

## RESULTS

### Demographic and household information

**Table 2** offers information about the sample, from both Bangladesh (*n* = 240) and India (*n* = 258). Similar numbers of girls and boys participated; the children from Bangladesh were slightly older (6.2 years) than those from India (5.6 years). In Bangladesh, on average children lived with 5.6 people in their household (σ = 2.0) and in India children lived with 5.8 people in their household (σ = 2.5). All participants came from a low-income background with limited access to resources (see **Table 3**). Despite a handful of differences, the communities were extremely similar. Many of the data collectors and facilitators, who were from these communities, explained that there were comparable experiences and lifestyles among people from the chosen communities. Notably, few participants had access to a radio (Bangladesh 1.3%, India 8.5%) or computer (Bangladesh 0.4%, India 1.9%) but many had a black and white or color television (Bangladesh 36.7%, India 86%).

### Exposure to the Intervention

When asked the vague question if they “participated in their community in any special activities in the last few weeks?” most intervention participants answered “yes,” (Bangladesh parents 97.5% and children 100%; India parents 74.3% and children 84.1%). The comparison group participants did not report participating in such activities (Bangladesh parents, 0.0% and children, 0.0%; India parents 7.0% and children, 11.3%).

As a manipulation check, parents and children were asked if the activities they participated in focused on you/your child (“me”) or the entire community/many children (“we”). Regardless the child’s intervention group, participants felt that the activities were about all of the community’s children. All the parents and over 80% of children in the “me” and “we” intervention groups felt the activities and content (focused on wearing sandals, latrine use, and handwashing) concentrated on children in general.

**FIGURE 1:**
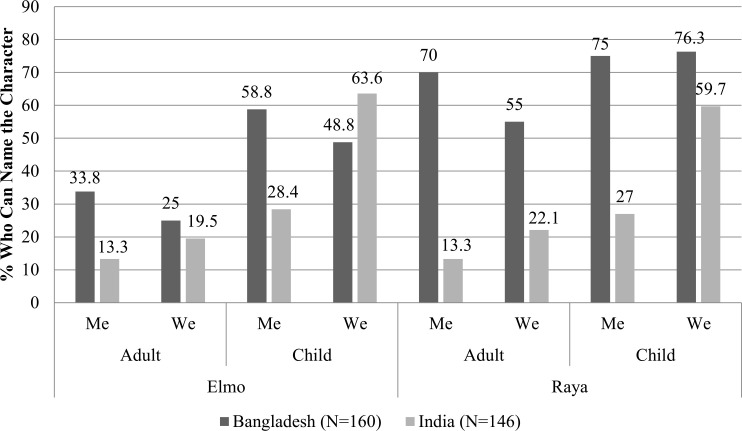
Percentage of intervention participants who could name Elmo and Raya in Bangladesh (*N* = 160) and India (*N* = 146).

In the post-intervention data, those in the “me” and “we” groups were familiar with the “*Cleaner, Healthier, Happier”* characters while those in the comparison group remained unfamiliar (awareness was at 0% before and after for this group). **Figure 1** shows parent and child’s awareness of Elmo and Raya (Bangladesh *n* = 160, India *n* = 146). Fewer parents were able to name the characters than children, with greater or equal recognition of the new character Raya compared to Elmo. There were large differences by intervention group, especially among children in India.

### Knowledge

**Table 4** presents the standardized knowledge change scores. For adult knowledge in Bangladesh the “me” and “we” group both had mean change scores greater than 1.0, indicating improvement in knowledge after the intervention compared to the mean change for comparison group participants (*p* < 0.001). In India there was no significant difference between groups.

**Table 5** shows the linear regression model for the intervention predicting a change in knowledge. In Bangladesh, change in adult knowledge increased on average 1.35 points for intervention participants (“me” and “we” groups combined) compared to the reference of the comparison group (possible scale range from 0 to 8) (*p* < 0.001). In both countries, change in child knowledge was not significantly predicted by the intervention.

**TABLE 5:** Linear regression predicting change in knowledge and attitudes from intervention (“me” and “we” combined) using comparison group as the reference.

Intervention predictor values	Bangladesh			India		
	β	95% CI	Sig.	β	95% ci	Sig.
Adult knowledge	1.35	0.15, 1.05	*p* < 0.001	0.42	−0.12, 0.96	ns
Child knowledge	0.18	−0.6, 0.41	ns	−0.22	−0.47, 0.03	ns
Adult general attitudes	−1.59	0.56, −2.68	*p* < 0.01	−0.06	−1.22, 1.11	ns
Child general attitudes	1.21	0.41, 2.01	*p* < 0.01	0.27	−0.69, 1.23	ns

*All models controlled for child age, child gender, child baseline knowledge, and parent education*.

### Attitudes

Looking at attitude scores in **Table 4**, we observed an increase for the parents in Bangladesh in the “me” and comparison groups, and a decrease for the “we” group (*p* < 0.01). There is a significant increase in child attitudes for both intervention groups, compared to the comparison group (*p* < 0.05). In India, all groups showed slight increases in child attitudes, but with no significant differences.

In the regression model for Bangladesh, adult attitudes decreased for intervention participants (“me” and “we” combined) compared to the comparison group (*p* < 0.01) while child attitudes increased for those in the intervention (*p* < 0.01) (see **Table 5**). In India, neither adult nor child attitudes was significantly predicted by the intervention.

### Behaviors

#### Latrine Use

Overall, as reported by parents, the percentage of children who defecated in the open at home decreased from before to after the intervention (in Bangladesh 48.3 to 42.1%; in India 3.9 to 0.9%). The amount of children using a ventilated improved latrine, as reported by parents, increased overall (in Bangladesh 10.8 to 24.6%; in India 44.2 to 74.4%).

**Table 6** shows that participants from Bangladesh and India had positive change scores for the “me” and “we” groups, with regard to use of an improved ventilated latrine at home (*p* < 0.001). In Bangladesh, both intervention groups were significantly higher than the comparison group. In India, the “me” group was significantly higher than the comparison and “we” groups. For type of toilet facility used at school, Bangladesh participants in the “me” and “we” groups compared to the comparison group showed significant increases (*p* < 0.05). In India, all groups showed some positive change, but the differences were not significant.

**Table 7** shows that participation in the intervention significantly predicted child latrine use at home [Bangladesh, *p* < 0.001; India (*p* < 0.05)]. In Bangladesh but not India, the change in use of an improved latrine at school was also significantly predicted by the intervention (*p* < 0.001).

**TABLE 7:** Linear regression predicting change in behaviors from intervention (“me” and “we” combined) using comparison group as the reference.

Intervention effects on predicting behavior change outcomes	Bangladesh			India		
	β	95% CI	Sig.	β	95% CI	Sig.
**Toileting**						
Improved toilet at home	0.30	0.17, 0.43	*p* < 0.001	0.20	0.01, 0.40	*p* < 0.05
Improved toilet at school	0.42	0.23, 0.62	*p* < 0.001	0.18	−0.09, 0.44	ns
**sandal/shoe wearing**						
Change in wearing shoes outside the house (parent response)	0.56	0.26, 0.87	*p* < 0.001	0.27	0.07, 0.48	*p* < 0.01
Change in wearing shoes when going to defecate (parent response)	0.19	−0.09, 0.46	ns	0.35	0.00, 0.71	*p* < 0.05
Change in wearing shoes last time at toilet (parent response)	0.12	−0.16, 0.40	ns	0.03	−0.30, 0.36	ns
**handwashing**						
Change in frequency immediately after defecation (parent response)	1.02	0.61, 1.42	*p* < 0.001	−0.11	−0.35, 0.13	ns
Change in soap frequency (parent response)	0.66	0.22, 1.10	*p* < 0.01	0.26	0.01, 0.51	*p* < 0.05
Change in soap frequency after defecation (parent response)	0.91	0.20, 0.52	*p* < 0.001	0.02	−0.25, 0.28	ns

*All models controlled for child age, child gender, child baseline knowledge, and parent education*.

#### Sandal and Shoe Wearing

As shown in **Table 6**, there were no significant differences in Bangladesh between the groups, but in India, the “we” group was significantly higher for children wearing their shoes “all the time” when going to defecate (*p* < 0.001).

The linear regression models indicate that change in wearing shoes outside the house was significantly predicted by the intervention in both countries [Bangladesh, *p* < 0.001; India (*p* < 0.01)] (see Table 7). Additionally, in India the intervention was a significant predictor of change for the outcome of always wearing shoes when going to defecate (*p* < 0.05).

#### Handwashing

In Bangladesh, all three of these variables showed significant increases in the intervention groups compared to the comparison group, as seen in **Table 6** (handwashing frequency immediately after defecation *p* < 0.01, soap frequency *p* < 0.001, soap frequency after defecation *p* < 0.001). The “me” group scored higher than the “we” group; however, the intervention group change scores were not significantly different from each other.

In India, the frequency of soap use when handwashing significantly increased for both intervention groups. The “we” group was slightly higher than the “me” group for all handwashing variables, but there were no significant differences between the intervention groups.

In the linear regression models for Bangladesh the intervention was a significant predictor for change in the frequency of children washing their hands immediately after defecation (*p* < 0.001) and frequency of soap use by children after defecation (*p* < 0.001) (see Table 7). The intervention was also shown to significantly predict frequency of child soap use in both countries [Bangladesh, *p* < 0.01; India (*p* < 0.05)].

## DISCUSSION

This study suggests that the *Cleaner, Happier, Healthier* media intervention improved hygiene knowledge, attitudes, and behaviors among parent and child participants in Bangladesh and India. While the research design limits our ability to make causal inferences, these findings offer that communities exposed to intervention activities and materials performed better in sanitation and hygiene measures. Target behaviors, such as using the latrine, wearing sandals, and handwashing, improved when examining change before and after the intervention.

The strength of this study is that we are confident that this media intervention successfully reached the participants, as we observed high recall and understanding of the presented and novel materials. Raya, a new Muppet^®^, who encourages latrine use, handwashing with soap, and sandal wearing, was well known by participants.

Interestingly, many participants were unable to discern the differences in intervention approaches, distinguishing the individual “me” versus community-based “we” focus. Participants in both intervention groups felt the focus of the intervention activities were directed toward improved health of *all* the community’s children. Most children and all the adults across groups perceived the intervention as a social good, and not about improving individuals’ health. There are several possible explanations. To begin, the distinction between social and individual orientations in South Asia may be less pronounced than it is in other countries. Culturally, people may think in a group and not personal orientation based on more collectivist traits of the may think of the group and not have a personal orientation, drawing from collectivist traits (Triandis et al., 1988). Contributing to this inclination, the interventions occurred in social and community settings rather than household settings, possibly contributing to participants viewing this as a social experience. From this work, we cannot tell if the intervention was too weak in presenting the “me” approach or if we were facing too strong beliefs from the communities. This is a small study, and we would be interested to see if “me” versus “we” approach shows differences in other locations. While the research team felt that developing and measuring the personal “me” and social “we” approaches were appropriate and this was approved by in-country project managers, future research should be cognizant of cultural differences in the conceptualization of WASH behaviors and be sure to engage the local community in the development of all major intervention components.

If this research had found consistent and significant improvements in knowledge, attitudes, and behaviors attributable to one approach, it would suggest that the health communication endeavors use “me” or “we” in message delivery. Based on this work, regardless of approach, children benefited from exposure to the overall intervention. This suggests that neither approach resulted in more gains, and either can be used. In that we observed conflicting results, we would recommend further research be conducted with additional interventions and samples.

Knowledge and attitudes were pooled and standardized across baseline and post to better allow for comparisons. Adult knowledge in Bangladesh, but not in India, showed significant increases in the intervention groups compared to the comparison group. Children’s knowledge did not change significantly in either country, but this may be due to a ceiling effect, as children started with solid knowledge and did not have much room for improvement. For attitudes, adult and children’s change scores were significantly different by group in Bangladesh. While children’s attitudes improved the most in the intervention groups, for adult attitudes the most positive change was in the comparison group, follow by the “me” group. The intervention was a significant predictor of parent knowledge and parent and child attitudes but only in Bangladesh. Adult general attitudes were actually predicted to decrease in the intervention group (“me” and “we” combined) compared to the comparison group. This may be due to lack of adult involvement with the intervention materials and messaging, as the program was targeted toward young children.

Participants in both countries reported significant gains in the targeted behaviors of latrine use, sandal/show wearing, and handwashing. Neither the “me” nor the “we” intervention group consistently outperformed the other. Also, there were other outcomes that were not impacted by the intervention in a significant way. The lack of differences between the “me” and “we” groups led to combining the intervention groups when conducting regression analyses. Participants in both groups were exposed to the new character Raya and similar intervention content, differing only in the focus on healthy behaviors benefiting the individual (“me” group) or the community (“we” group). The intervention (“me” and “we” combined) was a significant predictor of many targeted behaviors, including latrine use, sandal/show wearing, and handwashing.

An important strength of this research was that great care was taken to implement this study; local researchers were well-trained and instruments were designed with the young child in mind. Research instruments were designed by child development experts and were refined by in-country team members to ensure cultural and age appropriateness of materials. In-country researcher teams, along with NGO partners, carefully chose community-based workers to take the messages of the project to the children and community members. These program facilitators received multi-day training to implement the “*Cleaner, Healthier, Happier*” program with either a focus on social motives (the “we” group) or individual motives (the “me” group). In-country researchers all had prior experience working with children and were trained in data collection methods.

When interviewing children, pictures were used and researchers accepted pointing or a head nod as a correct answer. This was important in order to receive reliable answers from young children, who are unfamiliar with and might be hesitant to participate in the research process (Borgers et al., 2000). However, there is concern that both adult and child participants provided responses which they perceived the researchers wanted or expected to hear. This courtesy bias has been shown to occur in other hygiene interventions (Freeman et al., 2014). For example, practically all children said they washed their hands with soap after using the latrine, before and after the intervention. Adults and children reported high frequency of shoe wearing, even when the researchers observed that this was not the case. This casts a limitation on the reported results. However, a benefit of using change scores for analysis is that results focus on the improvements that have occurred rather than reporting positive behaviors that may have existed prior to the intervention. Even if the results in the above examples were due to inflated responses, because participants said they engaged in these behaviors before and after the intervention the change score was low, giving potentially conservative findings on intervention effects (Brown and Burrows, 1992). It is also possible that social desirability increased as participants gained knowledge on the importance of engaging in WASH behaviors. This could have led to inflated self-reports of positive WASH behaviors during the post-test.

Exploring the validity of all the responses was beyond the scope of this work; however, future research could require the data collection team to make observations in the communities and participants’ households around hygiene behaviors to gain a better understanding of response validity and future instruments should include additional measures of social desirability to account for this.

While it is possible that participants may have inflated some of their responses, employed approaches do allow for the assessment of the intervention’s impact. Manipulation checks were used so that on some questions it was not possible for participants to know material in advance of the intervention, such as baseline ability to name the new character Raya. In these cases no participants responded correctly at baseline; therefore, indicating all correct responses during follow-up were a result of knowledge gained from the intervention.

Another limitation is that this was not a randomized controlled trial. Researchers had hoped to randomize children into control versus the intervention groups; however, because the intervention was being delivered through the community (and also *via* schools in Bangladesh), it was unfeasible to randomly assign children to one group in these settings with a high degree of fidelity. Additionally, it was skeptical that in these slum locations contamination across groups could be controlled if randomization occurred within a community. It was also beyond this pilot to randomly assign enough communities to different groups, as it would have required 60 to 90 communities. As a result, in each country the interventions occurred in two separate but similar communities (one “me” and one “we” intervention group) and had a comparison group with a nearby, similar community.

This intervention lasted for 3 months and does not include research or information on long-term effects. It is possible that longer-term exposure would result in similar or even better outcomes. A sanitation and hygiene intervention in Zimbabwe showed greater improvements in handwashing and latrine use than previous short-term work by having a multi-year long education effort (Waterkeyn and Cairncross, 2005); however, a review of 27 sanitation studies showed no association with length of follow-up (Garn et al., 2017) so a long-term study should be conducted to determine if there are increased benefits to longer exposure. Sustainability of WASH interventions over time has shown to be difficult (Waddington and Snilstveit, 2009). It is possible that healthy sanitation and hygiene behaviors will continue; however, without additional studies we will not know if improved knowledge, attitudes, and behaviors associated with exposure to a media intervention persist. As well, several of the improved habits are dependent on an improved infrastructure and materials. The ability for children to use improved latrine facilities, wash hands, and wear shoes requires materials that are not always available and the success of health behavior interventions has been shown to increase by providing these infrastructure improvements (Garn et al., 2017). Infrastructure change is outside the scope of Sesame Workshop but studies have shown that even when infrastructure such as latrines are available, they often go unused without education focused on behavior change (Sanan and Moulik, 2007; Devine, 2009), which was the focus of this intervention. Results of this work suggest that children and adults who have received the *Cleaner, Healthier, Happier* interventions will be more informed and more likely to engage in healthier behaviors. This study showed significant gains among young children and their parents with a small intervention; future implementations of this program will likely continue to have positive effects on improving WASH knowledge, attitudes, and behaviors in resource poor areas.

## Footnotes

1 Items are reverse coded.

## References

[cit0001] Akter T., Ali A. R., and Dey C. N. (2014). Transition overtime in household latrine use in rural Bangladesh: a longitudinal cohort study. BMC Public Health 14:721. doi:10.1186/1471-2458-14-721 25022231 PMC4226980

[cit0002] Anderson D. R., Huston A. C., Schmitt K. L., Linebarger D. L., and Wright J. C. (2001). Early childhood television viewing and adolescent behavior: the recontact study. Monogr*.* Soc. Res. Child Dev*.* 66, I–VIII, 1–147. doi:10.1111/1540-5834.00120 11326591

[cit0003] Baker K. K., Dil Farzana F., Ferdous F., Ahmed S., Kumar Das S., Faruque A. S et al., . (2014). Association between moderate-to-severe diarrhea in young children in the Global Enteric Multicenter Study (GEMS) and types of handwashing materials used by caretakers in Mirzapur, Bangladesh. Am. J. Trop. Med. Hyg*.* 91, 181–189. doi:10.4269/ajtmh.13-0509 24778193 PMC4080560

[cit0004] Barnard S., Routray P., Majorin F., Peletz R., Boisson S., Sinha A et al., . (2013). Impact of Indian Total Sanitation Campaign on latrine coverage and use: a cross-sectional study in Orissa three years following programme implementation. PLoS ONE 8:e71438. doi:10.1371/journal.pone.0071438 23990955 PMC3749227

[cit0005] Bartram J., and Cairncross S. (2010). Hygiene, sanitation, and water: forgotten foundations of health. PLoS Med*.* 7:e1000367. doi:10.1371/journal.pmed.1000367 21085694 PMC2976722

[cit0006] Blumenthal D., DiClemente R., Braithwaite R., and Smith S. (eds) (2013). Community-Based Participatory Health Research: Issues, Methods, and Translation to Practice, 2nd Edn. New York: Springer Publishing Company.

[cit0007] Bonu S., and Kim H. (2009). Sanitation in India: Progress, Differentials, Correlates, and Challenges Asian Development Bank. Available at: http://hdl.handle.net/11540/6304">http://hdl.handle.net/11540/6304

[cit0008] Borgers N., de Leeuw E., and Hox J. (2000). Children as respondents in survey research: cognitive development and response quality 1. Bull. Methodol. Sociol*.* 66, 60–75. doi:10.1177/075910630006600106

[cit0009] Boschi-Pinto C., Velebit L., and Shibuya K. (2008). Estimating child mortality due to diarrhoea in developing countries. Bull.World Health Organ*.* 86, 710–717. doi:10.1590/S0042-96862008000900015 PMC2649491 18797647

[cit0010] Brown K., and Burrows C. (1992). How Should We Measure ‘Change’ in Utility Measures of Health Status: Or Should We? National Centre for Health Program Evaluation. Available at: http://citeseerx.ist.psu.edu/viewdoc/summary?doi=10.1.1.596.6086

[cit0011] Brown W. J. (2012). “Promoting health through entertainment-education media: theory and practice,” in The Handbook of Global Health Communication, eds Obregon R. and Waisbord S. (West Sussex, UK: John Wiley & Sons), 121–143.

[cit0012] Bryce J., Boschi-Pinto C., Shibuya K., Black R. E., and WHO Child Health Epidemiology Reference Group (2005). WHO estimates of the causes of death in children. Lancet 365, 1147–1152. doi:10.1016/S0140-6736(05)71877-8 15794969

[cit0013] Cole C. F., Labin D. B., and del Rocio Galarza M. (2008). Begin with the children: what research on “Sesame Street’s” international coproductions reveals about using media to promote a new more peaceful world. Int. J. Behav. Dev*.* 32, 359–365. doi:10.1177/0165025408090977

[cit0014] Dangour A. D., Watson L., Cumming O., Boisson S., Che Y., Velleman Y et al., . (2013). Interventions to improve water quality and supply, sanitation and hygiene practices, and their effects on the nutritional status of children. Cochrane Database Syst. Rev*.* 8, 1–99. doi:10.1002/14651858.CD009382.pub2 PMC11608819 23904195

[cit0015] Devine J. (2009). Introducing SaniFOAM: A Framework to Analyze Sanitation Behaviors to Design Effective Sanitation Programs Water and Sanitation Program Working Paper. Washington, DC: World Bank Available at: http://documents.worldbank.org/curated/en/272351468334778050/Introducing-SaniFOAM-aframework-to-analyze-sanitation-behaviors-to-design-effective-sanitationprograms

[cit0016] Engle P. L., Fernald L. C. H., Alderman H., Behrman J., O’Gara C., Yousafzai A et al., . (2011). Strategies for reducing inequalities and improving developmental outcomes for young children in low-income and middle-income countries. Lancet 378, 1339–1353. doi:10.1016/S0140-6736(11)60889-1 21944378

[cit0017] Esrey S. A., Potash J. B., Roberts L., and Shiff C. (1991). Effects of improved water supply and sanitation on ascariasis, diarrhoea, dracunculiasis, hookworm infection, schistosomiasis, and trachoma. Bull*.* World Health Organ*.* 69, 609–621. PMC2393264 1835675

[cit0018] Fewtrell L., Kaufmann R. B., Kay D., Enanoria W., Haller L., and Colford J. M. (2005). Water, sanitation, and hygiene interventions to reduce diarrhoea in less developed countries: a systematic review and meta-analysis. Lancet Infect*.* Dis 5, 42–52. doi:10.1016/S1473-3099(04)01253-8 15620560

[cit0019] Freeman M. C., Stocks M. E., Cumming O., Jeandron A., Higgins J., Wolf J et al., . (2014). Systematic review: hygiene and health: systematic review of handwashing practices worldwide and update of health effects. Trop. Med. Int. Health 19, 906–916. doi:10.1111/tmi.12339 24889816

[cit0020] Garn J. V., Sclar G. D., Freeman M. C., Penakalapati G., Alexander K. T., Brooks P et al., . (2017). The impact of sanitation interventions on latrine coverage and latrine use: a systematic review and meta-analysis. Int. J. Hyg. Environ. Health 220, 329–340. doi:10.1016/j.ijheh.2016.10.001 27825597 PMC5414716

[cit0021] Ghosh R. (2012). Child mortality in India: a complex situation. World J. Pediatr*.* 8, 11–18. doi:10.1007/s12519-012-0331-y 22282378

[cit0022] Guerrant R. L., Hughes J. M., Lima N. L., and Crane J. (1990). Diarrhea in developed and developing countries: magnitude, special settings, and etiologies. Rev. Infect. Dis*.* 12(Suppl. 1), S41–S50. doi:10.1093/clinids/12. Supplement_1.S41 2406855 PMC7792920

[cit0023] Gupta K. L., Arnold F. and Lhungdim H. (2009). Health and Living Conditions in Eight Indian Cities National Family Health Survey (NFHS-3), India, 2005-06. Mumbai: International Institute for Population Sciences; Calverton, Maryland, USA: ICF Macro Available at: http://rchiips.org/NFHS/sub_report.shtml

[cit0024] Huda T. M. N., Unicomb L., Johnston R. B., Halder A. K., Sharker M. A. Y., and Luby S. P. (2012). Interim evaluation of a large scale sanitation, hygiene and water improvement programme on childhood diarrhea and respiratory disease in rural Bangladesh. Soc. Sci. Med. 75, 604–611. doi:10.1016/j.socscimed.2011.10.042 22197292

[cit0025] Hueso A., and Bell B. (2013). An untold story of policy failure: the Total Sanitation Campaign in India. Water Policy 15, 1001–1017. doi:10.2166/wp.2013.032

[cit0026] Humphrey J. H. (2009). Child undernutrition, tropical enteropathy, toilets, and handwashing. Lancet 374, 1032–1035. doi:10.1016/S0140-6736(09)60950-8 19766883

[cit0027] Karanesheva T. (2015). Choosing the communication channel – a factor for effective health communication. Bulg. J. Public Health 7, 35–47.

[cit0028] Kibria N., and Jain S. (2009). Cultural impacts of Sisimpur, Sesame Street, in rural Bangladesh: views of family members and teachers. J. Comp. Fam. Stud*.* 40, 57–75.

[cit0029] Levine M. M., Kotloff K. L., Nataro J. P., and Muhsen K. (2012). The Global Enteric Multicenter Study (GEMS): impetus, rationale, and genesis. Clin. Infect. Dis*.* 55(Suppl. 4), S215–S224. doi:10.1093/cid/cis761 23169934 PMC3502311

[cit0030] Liu L., Li Q., Lee R. A., Friberg I. K., Perin J., Walker N et al., . (2011). Trends in causes of death among children under 5 in Bangladesh, 1993-2004: an exercise applying a standardized computer algorithm to assign causes of death using verbal autopsy data. Popul. Health Metr*.* 9, 43. doi:10.1186/1478-7954-9-43 21819600 PMC3160936

[cit0031] Mares M. L., and Pan Z. (2013). Effects of Sesame Street: a meta-analysis of children’s learning in 15 countries. J. Appl. Dev. Psychol*.* 34, 140–151. doi:10.1016/j.appdev.2013.01.001

[cit0032] Million Death Study Collaborators (2010). Causes of neonatal and child mortality in India: a nationally representative mortality survey. Lancet 376, 1853–1860. doi:10.1016/S0140-6736(10)61461-4 21075444 PMC3042727

[cit0033] Montgomery M. A., and Elimelech M. (2007). Water and sanitation in developing countries: including health in the equation. Environ. Sci. Technol. 41, 17–24. doi:10.1021/es072435t 17265923

[cit0034] Murthy G. V. S., Goswami A., Narayanan S., and Amar S. (1990). Effect of education intervention on deaecation habits in an Indian urban slum. J. Trop. Med. Hyg*.* 93, 189–193. 2348497

[cit0035] Nasrin D., Wu Y., Blackwelder W. C., Farag T. H., Saha D., Sow S. O et al., . (2013). Health care seeking for childhood diarrhea in developing countries: evidence from seven sites in Africa and Asia. Am. J. Trop. Med. Hyg*.* 89(Suppl. 1), 3–12. doi:10.4269/ajtmh.12-0749 PMC3748499 23629939

[cit0036] National Institute of Population Research and Training (NIPORT), Mitra and Associates, and ICF International (2016). Bangladesh Demographic and Health Survey 2014. Dhaka, Bangladesh/Rockville, MD, USA: NIPORT, Mitra and Associates, and ICF International. Available at: https://dhsprogram.com/pubs/pdf/FR311/FR311.pdf

[cit0037] Patel A., Koizumi N., and Crooks A. (2014). Measuring slum severity in Mumbai and Kolkata: a household-based approach. Habitat Int. 41(Supplement C), 300–306. doi:10.1016/j.habitatint.2013.09.002

[cit0038] Prüss-Ustün A., Bartram J., Clasen T., Colford J. M. Jr., Cumming O., Curtis V et al., . (2014). Burden of disease from inadequate water, sanitation and hygiene in low- and middle-income settings: a retrospective analysis of data from 145 countries. Trop. Med. Int. Health 19, 894–905. doi:10.1111/tmi.12329 24779548 PMC4255749

[cit0039] Ryan A. B. (2006). “Post-positivist approaches to research,” in Researching and Writing Your Thesis: A Guide for Postgraduate Students, eds Antonesa M., Fallon H., Ryan A. B., Ryan A., Walsh T., and Borys L. (MACE: Maynooth Adult and Community Education), 12–26. Available at: http://eprints.maynoothuniversity.ie/874/

[cit0040] Sanan D., and Moulik S. G. (2007). Community-Led Total Sanitation in Rural Areas: An Approach the Works Water and Sanitation Program: South Asia. Washington, DC: World Bank. Available at: http://documents.worldbank.org/curated/en/672891468324551045/Community-led-total-sanitation-in-rural- areas-an-approach-that-works

[cit0041] Sesame Workshop. (n.d.). Our Results. Available at: http://www.sesameworkshop.org/what-we-do/our-results/

[cit0042] Sommer M., Ferron S., Cavill S., and House S. (2015). Violence, gender and WASH: spurring action on a complex, under-documented and sensitive topic. Environ. Urban. 27, 105–116. doi:10.1177/0956247814564528

[cit0043] Sood S., Shefner-Rogers C., and Skinner J. (2014). Health communication campaigns in developing countries. J. Creat. Commun*.* 9, 67–84. doi:10.1177/0973258613517440

[cit0044] Triandis H. C., Bontempo R., Villareal M. J., Asai M., and Lucca N. (1988). Individualism and collectivism: cross-cultural perspectives on self-in group relationships. J. Pers. Soc. Psychol. 54, 323. doi:10.1037/0022-3514.54.2.323

[cit0045] UNICEF, and WHO (2015). “25 years progress on sanitation and drinking water: 2015 update and MDG assessment,” in Sustainable Development Goals: 17 Goals to Transform Our World. Geneva, Switzerland: WHO Press, United Nations Available at: http://www.un.org/sustainabledevelopment/sustainable-development-goals/

[cit0046] United Nations (2016). Sustainable Development Goals: 17 Goals to Transform Our World. Available at: http://www.un.org/sustainabledevelopment/sustainable-development-goals/

[cit0047] Vaillancourt P. M. (1973). Stability of children’s survey responses. Public Opin. Q. 37, 373–387. doi:10.1086/268099

[cit0048] Waddington H., and Snilstveit B. (2009). Effectiveness and sustainability of water, sanitation, and hygiene interventions in combating diarrhoea. J. Dev. Eff*.* 1, 295–335. doi:10.1080/19439340903141175

[cit0049] Waterkeyn J., and Cairncross S. (2005). Creating demand for sanitation and hygiene through Community Health Clubs: a cost-effective intervention in two districts in Zimbabwe. Soc. Sci. Med. 61, 1958–1970. doi:10.1016/j.socscimed.2005.04.012 15927329

[cit0050] Wright J. C., Huston A. C., Murphy K. C., St. Peters M., Piñon M., Scantlin R et al., . (2001). The relations of early television viewing to school readiness and vocabulary of children from low-income families: the early window project. Child Dev*.* 72, 1347–1366. doi:10.1111/1467-8624.t01-1-00352 11700636

[cit0051] Yusuf M., and Zakir Hussain A. M. (1990). Sanitation in rural communities in Bangladesh. Bull. World Health Organ*.* 68, 619–624. 2289297 PMC2393196

